# Localization within Hostile Indoor Environments for Emergency Responders

**DOI:** 10.3390/s22145134

**Published:** 2022-07-08

**Authors:** Alex Boyle, Matthew E. Tolentino

**Affiliations:** Intelligent Platforms & Architecture Lab, University of Washington, Tacoma, WA 98402, USA; boylea4@uw.edu

**Keywords:** IoT, localization, indoor navigation, sensors

## Abstract

Recent advances in techniques to improve indoor localization accuracy for personnel and asset tracking challenges has enabled wide-spread adoption within the retail, manufacturing, and health care industries. Most currently deployed systems use distance estimates from known reference locations to localize a person or asset using geometric lateration techniques. The distances are determined using one of many radio frequency (RF) based ranging techniques. Unfortunately, such techniques are susceptible to interference and multipath propagation caused by obstructions within buildings. Because range inaccuracies from known locations can directly lead to incorrect position estimates, these systems often require careful upfront deployment design to account for site-specific interference sources. However, the upfront system deployment requirements necessary to achieve high positioning accuracy with RF-based ranging systems makes the use of such systems impractical, particularly for structures constructed of challenging materials or dense configurations. In this paper, we evaluate and compare the accuracy and precision of alternative RF-based devices within a range of indoor spaces composed of different materials and sizes. These spaces range from large open areas such as gymnasiums to confined engineering labs of traditional buildings as well as training buildings at the local Fire Department Training Facility. Our goal is to identify the impact of alternative RF-based systems on localization accuracy and precision specifically for first responders that are called upon to traverse structures composed of different materials and configurations. Consequently, in this study we have specifically chosen spaces that are likely to be encountered by firefighters during building fires or emergency medical responses. Moreover, many of these indoor spaces can be considered hostile using RF-based ranging techniques. We built prototype wearable localization edge devices designed for first responders and characterize both ranging and localization accuracy and precision using alternative transceivers including Bluetooth Low Energy, 433 MHz, 915 MHz, and ultra-wide band. Our results show that in hostile environments, using ultra-wide band transceivers for localization consistently outperforms the alternatives in terms of precision and accuracy.

## 1. Introduction

One of the most frightening things for a firefighter to hear over the radio during a fire response operation is the word “Mayday”. This means a firefighter is lost, disoriented, or injured within a burning building and requires immediate extraction [1]. When such worst-case scenarios occur, the mission of the on-site responders shifts to rescuing the firefighter that called the Mayday. Even though firefighters are equipped with LED strobes, an audible alarm, and a hand-held radio to communicate, during many structure fires the environment encountered by responders renders these devices ineffective. As such, firefighters regularly train using protocols to maintain location awareness within a building. However, office buildings with complex floor plans or warehouses with floor-mounted machinery can still lead to disorientation, particularly in smoke-filled, zero-visibility conditions. Consequently, even though communication may be possible via radio, firefighters may not be able to accurately describe their location to enable rescuers to reach them quickly. Arming responding firefighters with an indoor positioning system could potentially save their lives be pinpointing a downed firefighter to rescue teams. There has been significant recent progress on techniques and technologies to improve indoor localization accuracy [2,3,4,5,6,7]. In general there are two leading approaches to localization that could be useful for first responders: pedestrian dead reckoning (PDR) and radio-frequency-based (RF) ranging using wireless transceivers. Pedestrian dead reckoning leverages inertial measurement units (IMUs) coupled with a micro-controller that translates motion along multiple axes to estimate position and trajectory by integrating accelerometer and gyroscope readings [8,9,10]. Unfortunately, this can also lead to increasing localization error over time due to accumulated error, while such drift errors can be reduced using filtering algorithms, this requires additional computational capacity to be useful in real-time. An alternative approach to indoor localization is to use RF-based ranging techniques. Many of these use the received power of wirelessly transmitted signals during communication to estimate distances from nodes positioned in known locations, while others leverage message propagation delays. Using such techniques, up to centimeter-level ranging accuracy is achievable. However, signal fading, reflection, and diffusion cause multipath effects that can significantly impact ranging accuracy in the presence of obstructions such as walls or other reflective building materials, while some previous studies have shown that localization accuracy can be improved with RF-hostile environment with significant calibration or through a priori characterization, such steps are impractical in a system used by first responders entering unknown spaces [11].

Given the potential of high location accuracy using RF-based devices for localization, our goal in this work is to characterize the ranging accuracy and consequent localization accuracy across a range of spaces that are regularly encountered during fire response operations. These include buildings with large, open spaces, as studied in previous work, as well as hostile indoor environments susceptible to signal reflections, refraction, and absorption. We have built prototype device nodes with five different transceivers that operate across the open frequency spectrum designated for industrial, scientific, and medical purposes (e.g., ISM bands) for our localization evaluation. These include Wi-Fi (2.4 Ghz), Bluetooth Low Energy (2.4 Ghz), 915 MHz, 433 MHz, and Ultra Wide Band (3.1–10.6 Ghz). Using these devices, our initial characterization focused on conducting simple linear ranging experiments with just two nodes to identify the ranging accuracy and precision for simple device to device ranging. Based on these results, we then conducted full localization experiments using three anchors and a single tag. While traversing each space, we continuously calculated our two dimensional position using geometric lateration. Because our goal is to be able to localize within unknown, potentially hostile indoor environments, these experiments focused on characterizing localization within three unique spaces including (1) an open gymnasium with minimal obstructions (minimal interference), (2) a mixed use space that included a passage way, open area, and multiple conference rooms (moderate interference), and finally (3) an engineering research lab outfitted with equipment, wiring, people, and exposed infrastructure (high interference). We then evaluated our indoor localization system with real firefighter hose teams during a live fire attack scenario within two additional indoor spaces at the local Fire Department’s Training Center. Based on these experiments, this paper makes the following contributions:

1. Two dimensional RF-based localization using Wi-Fi, BLE, 433 MHz, and 915 MHz in hostile environments is impractical, while techniques to cope with multipath effects even in large open areas may be helpful, these are insufficient to yield localization to enable the extraction of an injured or disoriented person in visibility-limited, hazardous conditions.
2. Simple linear ranging experiments are sufficient to demonstrate the deleterious impact of multipath effects on localization in two dimensional space using wireless transceivers. Our experiments revealed that among the five transceivers evaluated, distance estimates using 433 MHz and UWB scaled most consistently with actual distances.
3. Using UWB transceivers we were able to consistently and continually localize while operating within several hostile environments–including during live fire attack scenarios–with sub-meter accuracy.

## 2. Background

The proliferation of devices with integrated GPS receivers has enabled the real-time tracking of personnel, vehicles, and other assets. For fire departments, GPS-enabled devices have been incorporated into fire engines, ladders, and other vehicular apparatus to enable Fire Chiefs and Incident Commanders access to the arrival time of personnel to active response scenes. However, as firefighters are deployed to rescue trapped victims, deliver emergency medical aid, or extinguish fires, access to GPS signals is limited. To accurately track firefighters within buildings, a real-time indoor positioning system (IPS) is needed.

### 2.1. Ranging Devices and Techniques

There are many different types of indoor positioning systems, which vary based on devices and techniques. Many systems use pedestrian dead reckoning (PDR) involving the use of wearable inertial measurement units (IMUs) to detect and track directional movement and speed and incrementally update estimated positions using motion models. A key advantage of PDR systems is that minimal hardware and supporting infrastructure is required, enabling independent operation. Unfortunately, these systems suffer from accuracy and precision challenges due to accumulated drift [12,13,14,15].

An alternative approach is to leverage RF-based communication between multiple devices. Wireless localization systems incorporate transceivers that communicate on open access radio frequencies. For example, Wi-Fi and Bluetooth devices, which operate in the 2.4 GHz spectrum, have been used extensively due to widespread deployment of routers [16,17,18]. Transceivers in the sub-gigahertz range, particularly in the unlicensed 433 MHz and 915 MHz ranges, have been used as well [19,20,21]. Techniques such as ultrasound, which operates in the 20–22 kHz frequency range, just outside the range of human hearing, have been shown to be effective in some cases. Using radio-based communication for ranging ensures that objects, or in our case, personnel to be tracked retain the ability move freely within spaces.

To use wireless transceivers for localization, distances between multiple reference devices, each at known locations, must be determined; these distances can then be used to calculate a location within a space through geometric lateration. Several techniques can be commonly used to calculate these distances, including received signal strength (RSS), time difference of arrival (TDoA), and two-way ranging (TWR).

Received Signal Strength. Received Signal Strength (RSS) relates radio transmission power with received power at an endpoint [2,21]. If the transmit power level is known, the power actually received can then be used to estimate the distance between a transmitter and a receiver based on a path loss model. In the absence of interference, such as two transceivers operating within direct line of sight, the received power decreases by the square of the distance [22]. Within indoor environments, interference from nearby objects including scattering, reflection, diffusion, and refraction can significantly impact received power. Despite these challenges, most transceivers support measuring received power so this technique is commonly used in RF-based localization systems [23]. In general, received power is translated to distance through a path loss model derived from Equation (1).

(1) RSSI=−10nlog10(distance)+A

RSSI is the received signal strength indicator, expressed in dBm, while *n* is the signal propagation constant, and *A* is the reference received signal strength (in dBm) measured at a distance of one meter. The values of *n* and *A* must be determined empirically. Identifying and tuning the signal propagation constant is critical to localization accuracy within indoor spaces, as RSSI can vary widely depending on the materials and obstructions. However, when properly tuned, such as the case in fingerprinting, using RSSI as a proxy for distance via tuned path loss model can lead to a localization system with reasonable accuracy with minimal communication overhead.

Time Difference of Arrival. This technique uses a one-way message transmission from a device to be localized to estimate distances between devices mounted in fixed locations within a space. Two time stamps, one at message transmit time and second upon message receipt, are used to determine distance based on message propagation time. Devices using this approach require tightly synchronized clocks to determine distance. This often requires a secondary, wired communication channel to minimize clock drift, while communication overhead is minimal for ranging, the strict clock synchronization requirement increases infrastructure costs.

Angle of Arrival. When devices are equipped with an antenna array and high-precision timers, the angle wireless transmissions arrive can be determined using the time differences between antennas [21,24,25,26]. In such systems, the exchange of messages between multiple devices is not required as the direction of the sender can be determined by the receiver directly.

Two-Way Ranging. Another technique, referred to as two-way ranging (TWR), uses a series of time stamps captured when sending and receiving a sequence of small messages between devices to estimate the distance between devices. These message exchanges are often referred to as a ranging transaction. During each ranging transaction, time stamps are captured when each message is transmitted and received during the sequence of message exchanges. Using message send and receive times across multiple messages, the computational time required for sending and receiving devices can be isolated from the total message exchange time, even though the clocks on each device operate independently. This enables the total message transit time to be quantified across multiple exchanges, which can then be used to estimate the distance between two devices, alleviating the need for clock synchronization. To minimize message handling delays, ranging transaction messages are often incorporated into a layer-two (L2) network protocol.

### 2.2. Geometric Lateration

For localization, distances from multiple reference points, as well as the locations of those reference points, are used to calculate the position of an object. The unknown position is determined by the intersection of the reference points. In two dimensional space, this is referred to as trilateration. Although we use trilateration in this study, the lateration techniques used in this study to determine positions can be generalized to higher dimensions with the inclusion of additional reference points.

In trilateration, we determine our current unknown position based on knowledge of the location of three reference points and the current distance to those points. The distances *d*_1_, *d*_2_, and *d*_3_ between our current unknown position (*x*, *y*) and reference points (*x*_1_, *y*_1_), (*x*_2_, *y*_2_), and (*x*_3_, *y*_3_) can be determined based on Equations (2)–(4).

(2) $${\left(x-x_{1}\right)}^{2}+{\left(y-y_{1}\right)}^{2}=d_{1}^{2}$$

(3) $${\left(x-x_{2}\right)}^{2}+{\left(y-y_{2}\right)}^{2}=d_{2}^{2}$$

(4) $${\left(x-x_{3}\right)}^{2}+{\left(y-y_{3}\right)}^{2}=d_{3}^{2}$$

Given this system of quadratic equations, we first expand each. We then and subtract Equation (3) from Equations (2) and (4) from Equation (3) which yields Equations (5) and (6), respectively.

(5) $$\left(2x_{2}-2x_{1}\right)x+\left(2y_{2}-2y_{1}\right)y=d_{1}^{2}-d_{2}^{2}-x_{1}^{2}+x_{2}^{2}-y_{1}^{2}+y_{2}^{2}$$

(6) $$\left(2x_{3}-2x_{2}\right)x+\left(2y_{3}-2y_{2}\right)y=d_{2}^{2}-d_{3}^{2}-x_{2}^{2}+x_{3}^{2}-y_{2}^{2}+y_{3}^{2}$$

(7) $$x=\frac{\left|\begin{array}{cc} d_{1}^{2}-d_{2}^{2}-x_{1}^{2}+x_{2}^{2}-y_{1}^{2}+y_{2}^{2} & 2(y_{2}-y_{1})y\\ d_{2}^{2}-d_{1}^{2}-x_{2}^{2}+x_{3}^{2}-y_{2}^{2}+y_{3}^{2} & 2(y_{3}-y_{2})y \end{array}\right|}{\left|\begin{array}{cc} 2(x_{2}-x_{1})x & 2(y_{2}-y_{1})y\\ 2(x_{3}-x_{2})x & 2(y_{3}-y_{2})y \end{array}\right|}$$

(8) $$y=\frac{\left|\begin{array}{cc} 2(x_{2}-x_{1})x & d_{1}^{2}-d_{2}^{2}-x_{1}^{2}+x_{2}^{2}-y_{1}^{2}+y_{2}^{2}\\ 2(x_{3}-x_{2}) & d_{2}^{2}-d_{1}^{2}-x_{2}^{2}+x_{3}^{2}-y_{2}^{2}+y_{3}^{2} \end{array}\right|}{\left|\begin{array}{cc} 2(x_{2}-x_{1})x & 2(y_{2}-y_{1})y\\ 2(x_{3}-x_{2})x & 2(y_{3}-y_{2})y \end{array}\right|}$$

For simplicity, we represent Equations (5) and (6) in matrix form. We find the *x* and *y* coordinates of the unknown position by solving Equations (5) and (6) using Cramer’s rule as shown in Equations (7) and (8). For each coordinate, we first find the determinant of the coordinate *x*, or *y*, respectively, and divide each determinant of the coordinate by the determinant of the matrix form of Equations (5) and (6).

## 3. Related Work

There has been considerable work to improve localization for indoor positioning systems [5,6,27,28,29]. For example, the widespread deployment of multiple Wi-Fi routers mounted within buildings to provide network connectivity to occupants has motivated the use of received signal strength (RSS) for localization [2,3,4,30,31,32,33,34,35,36,37,38]. The proliferation of Wi-Fi equipment makes these approaches attractive, but tend to suffer from multipath issues due to signal fading, reflection and diffusion that make high accuracy localization impractical, while fingerprinting techniques help improve accuracy using Wi-Fi, the required a priori configuration of such systems, or empirical determination of path loss model for a given space, limits their use in spaces within which the configuration and materials are unknown [39]. Alternatively, localization using Bluetooth transceivers has also gained traction due to the widespread deployment of BLE-enabled devices [23,40,41,42,43,44]. Similar to Wi-Fi, BLE-based localization systems typically use received signal strength as a proxy for distance, requiring customized path loss models for different spaces to meet accuracy requirements. Due to the noise in BLE receive signal strength, such systems tends to be more amenable to proximity-based localization. In contrast to using Wi-Fi and BLE devices operating in the 2.4 GHz spectrum, other localization systems have leveraged transceivers operating in the 915 MHz and 433 MHz frequency ranges [27,28,38]. Still others have used Ultra-Wide Band transceivers that operate between 3.1 GHz and 10 GHz [45].

Numerous indoor localization systems have been been previously proposed for use by firefighters during emergency events [7,34,46,47,48,49,50,51,52,53,54]. An early example was the Precision Personnel Location (PPL) system, designed specifically to track firefighters on the fireground [55,56,57]. This system leveraged multi-carrier wide band signals across a 150 MHz frequency spread. They used time difference of arrival (TDoA) to localize on-scene firefighters in two or three dimensions. These systems required the placement of large devices on all sides of a building to localize personnel within. This approach proved to be cumbersome and did not work well in large buildings, where received signals had to penetrate deep within unknown materials to accurately localize interior personnel. Later versions integrated inertial measurement units and Bayesian fusion techniques to generate pedestrian dead reckoning-based positions for improving localization accuracy within spaces that RF signals could not reach. The GLANSER system integrates foot-mounted inertial measurement units with filtering algorithms to estimate and display the positions of all fire response personnel on-scene to the Incident Commander [58,59]. Later versions of this system were reported to leverage ultra-wide band transceivers for ranging, although accuracy was limited to three meters. The FIREGUIDE system was developed to use RFID, Bluetooth, and Wi-Fi or cellular network connectivity to localize firefighters within buildings [49]. This system was developed to localize firefighters within structures and help them find the closest point of egress during interior fire response operations. Other systems have also been developed over the past ten years using alternative transceivers including UWB, Wi-FI, Bluetooth, ultrasound, and RFIDs [34,46,47,48].

While numerous systems have been developed to tackle the problem of tracking firefighters during emergency events, significant gaps remain. Two key requirements identified by the fire service as needed for any fire response localization system is accuracy within one meter, and sustained accuracy within any type of structure [46]. Most previously developed systems are unable to realize this level of accuracy without extensive a priori analysis of the indoor space to develop customized path loss models. These requirements are unrealistic given firefighters must respond to events at any building, any time. Moreover, most previously developed systems have not been evaluated with actual firefighter teams under realistic conditions faced during real events. These conditions, such as deployment of large volumes of water within buildings, can have a significant impact localization accuracy and precision. Additionally, most localization studies evaluate systems within indoor spaces with minimal electromagnetic interference.

Similar to previous work, we start by considering commonly used transceivers that operate in the open ranges of the frequency spectrum as well as localization techniques. Based on accuracy and precision analysis we then proceed to evaluate two localization systems within different types of spaces first responders would likely encounter. We then evaluate our localization system with active firefighter hose teams at their training facility.

## 4. Indoor Localization Devices and System

We built five versions of a minimal, portable localization system consisting of small, inexpensive battery-powered devices to record ranging data and calculate positions in a 2D Cartesian plane. We intentionally limited this study to two dimensions to enable comparisons with a large body of previous work and to actively work with firefighters responding to large, single-story commercial buildings such as warehouses that often result in mayday scenarios. Our ultimate goal was to identify a low-cost, small form factor that could be wearable by first responders. To minimize cost, we constructed devices composed of commodity off-the-shelf components using different transceivers for ranging. Our initial device designs incorporated Raspberry Pi3 devices, but during initial testing we found that these were large, cumbersome, and required large batteries to run for extended periods. Moreover, in some cases, particularly with ultra-wide band devices, we were not able to consistently meet timing requirements for time of flight measurements given the extensive software stack. Instead, we focused on using simpler, low-power Arduino, or Arduino-compatible, micro-controllers to create small, lightweight devices with a minimal software stack, yielding fine-grained and deterministic control over timing operations. We plan to consider using more robust embedded platforms with higher clock frequencies to enable on-device position calculations in the future.

### 4.1. Tags vs. Anchors

Devices used for localization typically operate in one of two roles. Devices, mounted in predetermined, fixed locations are usually referred to as anchors. Similar to previous work, we have configured anchors with constant 2D coordinates in a Cartesian plane representation of the space. Each anchor is configured to periodically broadcast messages to advertise their existence and position to listening devices. In our system, multiple anchors are distributed throughout indoor spaces, at specific measured locations, to ensure tags maintain connectivity to a minimum of three anchors to localize in 2D.

Devices that are expected to move within a space, such as those worn by firefighters, are referred to as tags. Tags are configured to listen for messages broadcast by anchors. Once tags have received messages containing position and distance information from a sufficient number of anchors, the position of a tag can be determined through trilateration. For ranging protocols that only require the transmission of a single message from anchors, tags simply need to receive messages from anchors, decode the source’s location, and then determine the range based on received power or differences in transmit and receive time stamps. In other ranging protocols, such as two-way ranging, that require bidirectional communication between tags and anchors, tags typically drive the necessary message exchange to determine the range.

In our system, any device can be configured to operate as a tag or anchor. The distinction between the two roles is managed in the firmware on each device. Identification numbers were assigned to each device to differentiate between devices beyond differences in x-y coordinates. Finally, in this evaluation, we did not compute the trilateration step on the tags themselves. Given the limited computational capacity of the devices, after a set of new ranges from three anchors was acquired, these were transmitted to a server over Wi-Fi to compute and store the tag’s location during experiments.

### 4.2. Ranging Devices

We assembled and tested five unique devices consisting of different transceivers and approaches to estimate distances between devices. Note that all devices, except for the BLE-only configuration, were configured with a Wi-Fi transceiver that enabled all devices to transmit data to a server for data collection and localization. This minimized the need for a display or local storage on each device. This section describes the composition of devices we evaluated.

BLE Ranging Devices. Our first positioning system focused on using devices with integrated 2.4 Ghz Bluetooth Low Energy transceivers. For these experiments we used a commercially available device called the LightBlue Bean from PunchThrough. Each Bean consists of an ATmega328p directly connected to a TI CC2540 SoC over UART. The TI CC2540 includes a complete BLE software stack in a form factor that measures 1.8″ by 0.8″ and is powered by a 2032 coin cell battery. The ATmega328p enables the use of standard Arduino sketches for control and communication. We developed and integrated custom firmware for the Bean which we flashed over BLE to enable each Bean to operate as either an anchor or tag. When a Bean operated as an anchor, it was configured to periodically broadcast beacon messages with Cartesian coordinates of its location embedded within. Each tag was configured to listen for these anchor messages, measure the received signal power upon receipt, and then translate received power estimate to distance using Equation (1).

Wi-Fi Ranging Devices. Numerous previous studies have leveraged Wi-Fi devices for ranging, although most have focused on leveraging fixed-mount routers. In our study, we built devices using the Arduino Uno, which consists of a 16 MHz ATmega328p micro-controller, and an Espressif ESP8266, which consists of a 32-bit Tensilica-based CPU, 2.4 Ghz radio transceiver compliant with 802.11 b/g/n W-Fi standards, and support for a TCP/IP stack. We used the same ranging protocol as the BLE-based device, by using the Arduino ATmega328p micro-controller to drive transactions directly over the ESP8266’s Wi-Fi transceiver, by-passing the embedded CPU. For devices specified to operate as anchors, the ESP8266 was configured to operate in Soft Access Point (AP) mode. Consequently tags were configured to connect to access points (e.g., anchors) and messages were periodically broadcast from anchors to tags. As part of receiving messages, the received power measured by the ESP8266 transceiver was translated into a distance based on radio propagation Equation (1).

433 MHz and 915 MHz Ranging Devices. We also built wearable tag and anchor devices using discrete 433 MHz and 915 MHz transceivers. These devices consisted of 16 MHz ATmega328p-based Arduino Uno microcontrollers coupled with SemTech SX1231 transceivers connected over SPI. We constructed one set of devices with the transceiver configured to operate at 433 MHz and a second set to operate at a frequency of 915 MHz. We used rigid commercial SMA-connector antennas designed specifically for each operating frequency range. By reducing the frequency, relative to Wi-Fi or BLE, we limit the available bandwidth on the channel, but gain increased range between devices. For the ranging protocol on these devices, we used a similar approach of having the anchors periodically broadcast messages with their Cartesian coordinates. Tags would listen for these messages and then upon receiving them use the received power estimates during the received transaction as a proxy for distance from the broadcasting anchor.

Ultra-Wide Band Ranging Devices. The last set of devices we assembled included Ultra-Wide Band radios. These devices were inspired by the high ranging accuracy and precision results that were reported as part of the Microsoft-sponsored indoor localization competition [60]. Similar to devices used within several years this competition, our devices were composed of 16 MHz ATMega328p-based Arduino Uno micro-controllers, ESP8266 for Wi-Fi connectivity to the localization server, and a Decawave DWM1000 transceiver module with integrated antenna connected via SPI bus. Unlike the other narrow band transceivers, UWB radios operate at greatly reduced power levels distributed across a wide frequency range with a specified center frequency. In our systems, we have configured the UWB to operate at a center frequency of 6.5 Ghz with a total throughput of 6.8 Mb/s. We used an open source DWM1000 Arduino library to configure each device to operate as a tag or anchor. Included in this library was an implementation of the standard 802.15.4 MAC protocol designed for two-way ranging, which we extended to enable the transmission of anchor position information similar to our other devices.

Unlike the other transceivers evaluated in this study, estimating the distance between UWB-based devices was not based on using received power as a proxy for distance. Instead, we used a two-way ranging protocol that involves devices exchanging a set of messages to determine the distance based on the time it takes for messages to propagate. This involves maintaining a set of high resolution time stamps to measure when a message is sent from transmitting devices as well as when a message is received on the receiving device. These time stamps are taken within the UWB transceiver to minimize interconnect delays between a transceiver and CPU within a system. Because the time stamps between devices can vary significantly, multiple messages must be exchanged. Minimally, three messages are required, although the protocol we used included a fourth message to report the calculated ranges determined at each anchor back to the tag. However, given our experiments were not focused on scaling the number of devices to be tracked, the additional overhead of the fourth message did not impact our results.

### 4.3. Localization Server

Given the ranges to a set of anchors and the Cartesian location of those anchors, we used an on-site web server to calculate the position of a tag. For this, we built a simple REST API that we used to serve POST and GET requests when new ranges between anchors and tags were acquired. Upon receipt the server would use the three anchor coordinates and new distances to estimate the position of the tag. This position was rendered on a local screen during experiments to verify operational functionality, but also recorded into a log for later analysis. We directly connected the server to a router we used to ensure Wi-Fi connectivity of devices for experiments within buildings.

## 5. Materials and Methods

Our goal in this study was to characterize the efficacy of leveraging existing indoor localization devices to build a system capable of operating in the harsh conditions faced by firefighters during structural fires. We started by assessing the accuracy and precision of the ranging devices described in the last section within unobstructed indoor spaces. We then proceeded to evaluate devices with the highest accuracy within increasingly hostile indoor environments. It should be noted that we did not initially consider UWB-based devices; they were added to the experimental design later after we had obtained initial results from all other devices.

### 5.1. Linear Scaling

Our first experiments included a straight forward linear scaling test to validate the estimated distances between two transceivers coupled with ranging protocol scaled proportionally with measured distances. These experiments were conducted in a large open, conference room without obstructions. For these, we placed one anchor device on a fixed pedestal elevated to a height of one meter. We then measured and marked linear distances from the anchor in one meter increments out to 30 m and configured a second device to operate as a tag. During each experiment we moved the tag away from the anchor in one meter increments and recorded a minimum of 20 distance measurements at each fixed location. For all devices, except UWB, we also recorded the raw received power measurements in decibel milliwatts (dBm) given the distance measurements were based on these power estimates. For UWB, we recorded the estimated distances based on two-way ranging using time of flight (ToF). We repeated each linear scaling experiment for every device configuration a minimum of three times and then computed the average distance and variance for each distance measurement.

Our goal in these experiments was two-fold. First, we wanted to validate the received power estimates used changed proportionally with distance. If the received power does not change consistently with distance, it would be difficult to discern accurate distances with any path loss model. Moreover, combining multiple incorrect ranges to calculate a 2D position using trilateration would be problematic due to the aggregated error. Second, accurately characterizing path loss under wide-ranging environmental conditions is difficult. So, in this work we recorded the raw decibel-milliwatt (dBm) values and then estimated distance using alternative path loss formulae to characterize the impact on a positioning system.

### 5.2. Localization within Indoor Spaces

**Figure 1:**
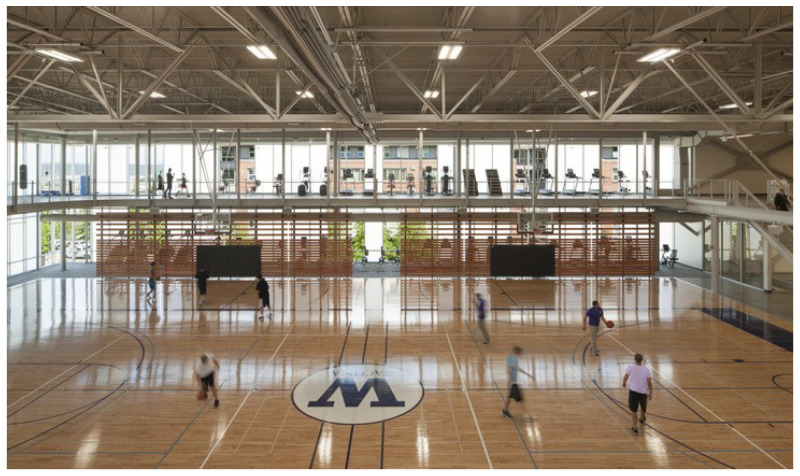
Photo of open gymnasium at YMCA at University of Washington that was used for range accuracy experiment as well as localization accuracy testing and characterization.

**Figure 2:**
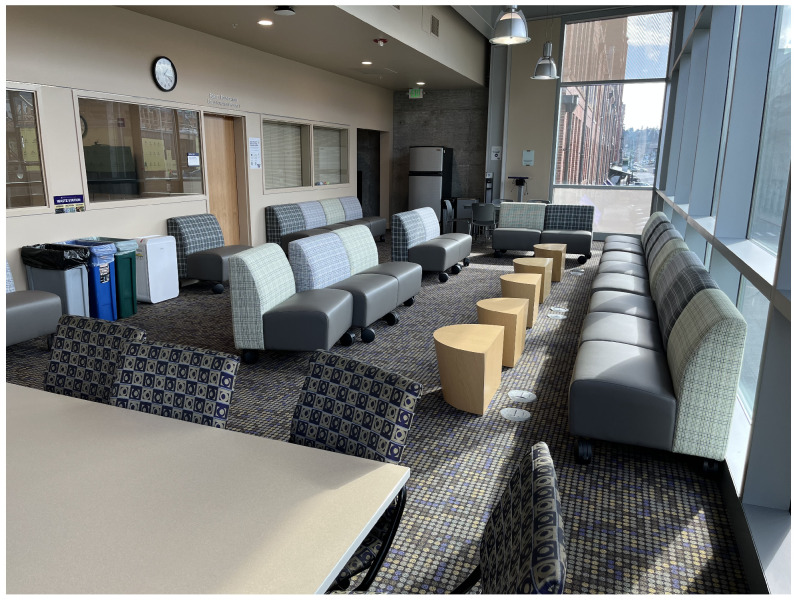
Photo of mixed use space at University of Washington that was used for range accuracy experiment as well as localization accuracy testing and characterization.

**Figure 3:**
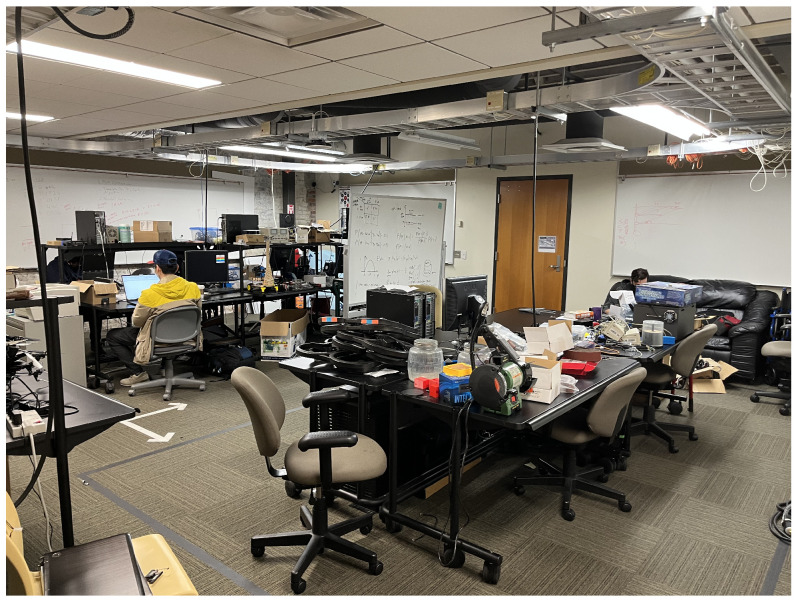
Photo of small engineering lab that was used for range accuracy experiment as well as localization accuracy testing and characterization. The black tape shown on the floor constituted one of the paths that was repeatedly used during the experiments discussed in this paper.

After the initial linear scaling experiments, we combined three anchors and a tag for a simple indoor 2D position system. Each tag was configured to range with the three anchors and use the three ranges to determine position via trilateration. These positioning experiments were initially conducted within three indoor spaces. These spaces included a large, open gymnasium as shown in Figure 1, a mixed-use space that included an open space, hallways and conference rooms as partially shown in Figure 2, and finally an electrical and computer engineering research lab populated with people as well as exposed wiring and active, powered equipment as shown in Figure 3. The gymnasium consisted of wooden floor, walls consisting of metal framing and glass, and a ceiling with large, exposed metal trusses. The mixed-used space consisted of large glass-wall, industrial carpet over a concrete floor, and drywall-sheathed metal framing typicaly of commercial buildings. The engineering lab also included carpet-covered concrete floors with embedded power lines, exposed metal framing along the ceiling containing large runs of power delivery cabling, and drywall sheathed metal framed walls. Our goal is to ensure our experiments replicated approaches conducted in large open spaces as in previous work, but also consider more hostile environments, subject to multipath signal propagation, that would likely be encountered by firefighters and be challenging for RF-based ranging systems due to obstructions and electromagnetic interference.

In each of the spaces, we placed three anchors in known locations at the periphery of the respective space that we carefully measured a priori. We then created a specific path through the space by placing tape on the ground. We then measured the locations of all anchors and the path using both a tape measure and a laser range finder. By measuring the tape path, we captured the ground truth path to use in the accuracy and precision analysis of calculated positions. Each anchor was then configured with its respective Cartesian coordinates before starting the tag. To ensure consistent runs for each device, we placed the tag on a cart that was navigated along the taped route and measured the transit time for each leg to ensure each run had approximately the same number of calculated positions. This was conducted similarly to the way the 2019 IPIN Indoor Localization Competition evaluation runs were conducted [61]. We repeated these experimental runs within each space five times.

### 5.3. Localization within Fire Training Buildings

Because our ultimate goal is to localize firefighters within hostile indoor environments, we expanded our initial experimental design to include spaces under conditions often faced by real firefighters.

**Figure 4:**
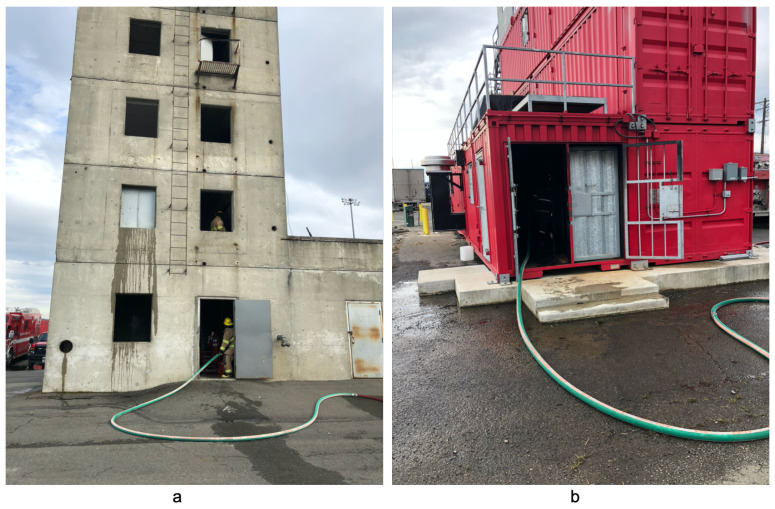
(**a**) Photo of concrete building used for live-fire experiments with Fire Department teams. (**b**) The second building was composed of multiple stacked corrugated metal shipping containers.

To do this, we collaborated with the local fire department at their training facility where they train new recruits as well as hold live fire response exercises. One of these buildings was a solid concrete, five story tower as shown in Figure 4. The second building was composed of multiple stacked corrugated metal shipping containers shown in Figure 4.

For these two spaces, we positioned the anchors on a single floor of the building. Within the concrete tower, we used the second floor and in the metal building we used the first floor. We then outfitted a three-man hose team with a single tag and asked them to traverse each of the spaces as they would during a live fire. This included navigating the space with a charged fire hose, delivering water to the space as if they were attacking a live fire. After learning the path they were trained to use while attacking a fire in such a space, we marked and measured their path to establish the ground truth path. During each run, the fire team would fully saturate every surface within the space with water, including the ceiling, all walls, and floor, making for an extremely wet environment. To ensure continued operation of our prototype devices, we enclosed all devices and batteries within clear, resealable bags to ensure continual operation during the experiments. Similar to our previous experiments, the fire team traversed each of the two spaces five times.

### 5.4. Metrics

Numerous metrics have been used to characterize indoor localization systems [4]. These include accuracy, precision, cost, complexity, robustness, and scalability. In this evaluation, we are focused on how to effectively locate first responders in grave danger. Consequently, the two primary metrics we consider are accuracy and precision, although in future work we plan to consider other factors. Accuracy refers to the mean Euclidean distance error between estimated positions and true positions. For most results we initially used average accuracy while traversing the designated path. In our later experiments using UWB we also compare results using the third quartile of calculated positions to enable comparisons in recent work [62]. We use precision to refer to the consistency of estimated positions while traversing a path. This is critical because based on the ranging technique used, there can be significant variance in estimated positions, while mitigating this variance has been a consistent topic in previous work, in this evaluation, we consider the accuracy and precision of raw, unfiltered estimated positions. Our choice of devices is motivated by the remaining metrics including cost, complexity, and form factor as any devices used would have to be carried by firefighters. However the primary focus of this work is to assess the accuracy and precision of the transceivers to build a localization system for first responders.

## 6. Results

### 6.1. Linear Ranging

Our initial experiments focused on characterizing the ranging accuracy of alternative RF transceivers using received signal strength as a proxy for distance. These first experiments were conducted in the same, open space to minimize the impact of multipath effects. Ideally, the estimated distance should be consistent with the measured distance. In terms of localization, this means that as the distance between a tag and any anchor changes, as would be the case when traversing a space, our updated Cartesian position estimate accurately reflects our current location using newly estimated ranges. If the estimated range between two devices does not scale consistently, using multiple devices to determine positions in two or more dimensions would be impractical given the combined error.

**Figure 5:**
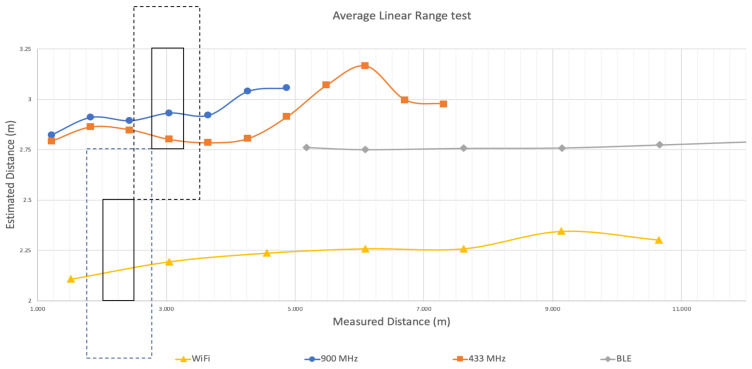
Estimated distance relative to measured distance between two devices using BLE, 433 MHz, 915 MHz, and Wi-Fi transceivers. Ideally, the estimated distances would scale proportionally with measured distances for each of the transceivers used. However, these results show that distance estimation using these transceivers with lateration techniques that require multiple ranges will lead to significant position accuracy error.

Figure 5 plots the estimated distances between one anchor and one tag as calculated at the tag using Wi-Fi, BLE, 433 MHz, and 915 MHz relative to measured distances. The *x*-axis constitutes real distance in meters and the y-axis shows the estimated distances calculated by translating received power at the tag to distance via Equation (1). As we moved the tag farther away from the anchor, we did see a decrease in the received power across all transceivers. However, when translated to distance, the results were inconsistent with actual distances across all transceiver types. For example, at 1.5 m, our estimated distance was 2.1 m for Wi-Fi, a difference of 0.6 m in accuracy. At 10.66 m, the estimated distance using Wi-Fi was 2.29 m, an error of 8.37 m. For BLE we observed similar accuracy limitations. At 5.18 m apart, the estimated distance between the anchor and tag was 2.76 m, a difference of 2.42 m. At 10.66 m, the estimated distance was only 2.77 m, for a difference of 7.89 m. Given the widespread use of Wi-Fi and BLE, we expected a closer correlation of RSSI to distance. However, as shown in Figure 5, using both Wi-Fi and BLE, there was only minimal change in estimated distance as we moved away from the anchor, leading to increasing error of up to 8 m at 10 m.

Similar to Wi-Fi and BLE, we also observed differences in estimated distances as we moved the tag farther away from the anchor using received power with the 433 MHz and 915 MHz transceivers. Using 915 MHz, the estimated distance was 2.82 m at an actual distance of 1.21 m, an error of 1.6 m. However, at 5.48 m, the estimated distance was 2.93 m, an error of 2.55 m. For 433 MHz, the results were similar. We started with the tag close to the anchor at only 0.69 m. At 0.69 m, the average estimated distance was 0.11 m, amounting to an error of 0.58 m. However, increasing the distance between the anchor and tag to 4.26 m, the distance was estimated at 0.51 m, an error of 3.75 m. When two devices were in extremely close proximity the error was minimized and estimated distances were either close to or within our precision target of one meter. However, the error in distance estimates increased as the actual distance increased. Moreover, the precision was notably lower as well.

The limited accuracy of ranging at increased distances can lead to position uncertainty. For localization, because we must combine multiple ranges to calculate a position, ranging precision between devices must be consistent to realize accurate positioning within a given precision target. Given this, we also analyzed the linear ranging results for precision at one meter (minimum precision requirement) and 0.5 m (preferred precision requirement). Figure 5 includes three windows that highlight the distances at which our estimated distances met these precision requirements. The boxes with dotted lines show the experiments where we observed one meter precision and the boxes with the solid lines show when ranging precision was within 0.5 m. It should be noted that BLE, shown as the gray points in Figure 5 did not meet our precision requirements at any distance. For Wi-Fi, when the devices were closer than three meters, our precision requirements were met. However, precision was unacceptably lower greater beyond three meters. Both 915 MHz and 433 MHz met our one meter precision requirement between 2.5 and 3.5 m from the anchor, and met the 0.5 m precision requirement between 2.75 and 3.25 m. This means that when multiple ranges are used to determine position, to maximize location precision, anchors should be located within the window of precision from the tag at any time. However, given many spaces this may not be practical.

### 6.2. Signal Propagation Analysis within Alternative Spaces

**Figure 6:**
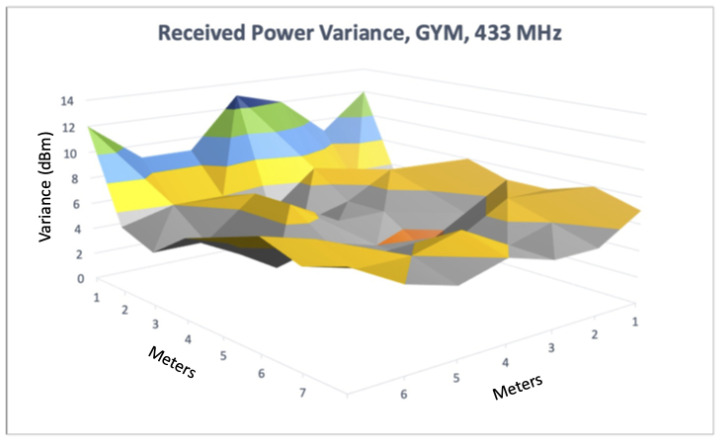
Received signal variance using 433 MHz transceivers in the open Gym space. Received signal power levels were collected at one meter increments within the space that contained the path we used for localization experiments.

**Figure 7:**
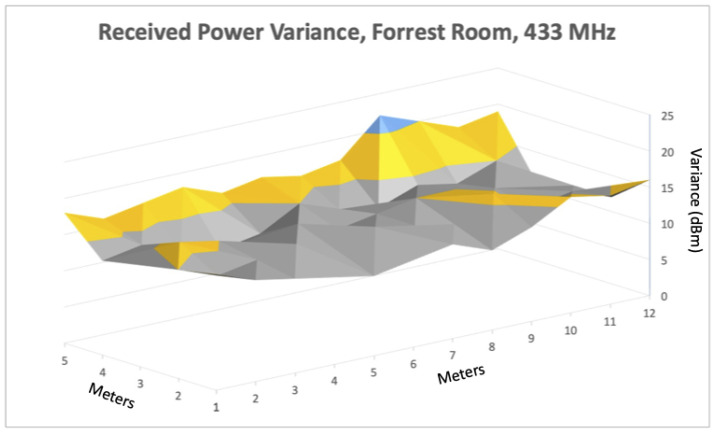
Received signal variance using 433 MHz transceivers in the mixed use, Forrest Room space. Received signal power levels were collected at one meter increments within the space that contained the path we used for localization experiments.

**Figure 8:**
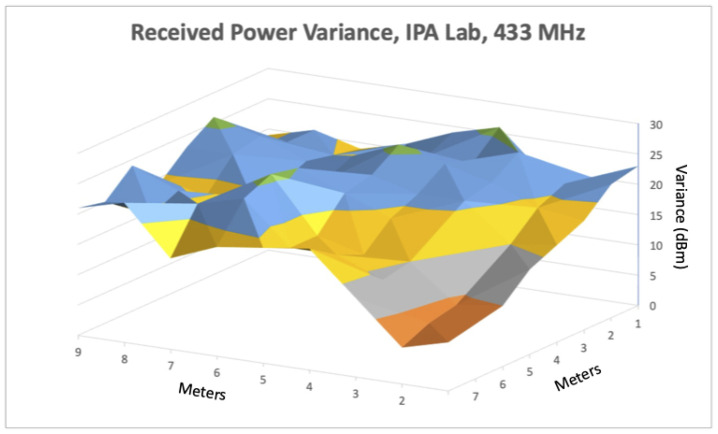
Received signal variance using 433 MHz transceivers in the IPA engineering lab. Received signal power levels were collected at one meter increments within the space that contained the path we used for localization experiments.

Our next set of experiments involved setting up a simple localization system that used ranges from anchors in fixed locations to calculate positions while traversing different spaces. As discussed in the previous linear ranging section, we observed better ranging accuracy using the 433 MHz transceivers compared to Wi-Fi, BLE, or 915 MHz. Based on that evaluation, and despite the observed precision limitations, we used the 433 MHz devices to localize within three distinct indoor spaces. These environments constitute a range of spaces representative of commercial spaces typically encountered by firefighters as well as an engineering lab. We used a large, open gymnasium within the local YMCA, a common study area with attached conference rooms called the Forrest Room, and a confined research lab (referred to as IPA research lab) populated with occupants and electronic equipment. Before conducting full localization experiments, we first profiled each space to characterize the signal fading within each space using the 433 MHz transceivers. We did this by placing a single transmitting device in a fixed location within each space and measured the received signal power in a grid pattern at one meter increments within the area that contained our ground truth path. For each point in the grid, we recorded multiple power measurements, and quantified received power variance at each point. Given our localization experiments use received power as a proxy for distance this characterization directly impacts range estimates, and hence our localization results. Figure 6, Figure 7 and Figure 8 show how received power varied within each space. In Figure 6, we see some dBm variance in the space, although it remains fairly consistent except near the wall, which aligns with our earlier linear ranging results. We observed higher variance in the Forrest Room than the Gym, with dBm variance highest nearest the corner. Furthermore, the variance in received power was highest within the IPA engineering lab with slightly lower variance nearest the transmitting device (e.g., the closest corner), due to direct line of sight placement. Overall, these results align with our earlier linear ranging results using 433 MHz and demonstrate that different space configurations, materials, and types of obstructions result in different signal fading characteristics.

### 6.3. Localization within Indoor Spaces

**Figure 9:**
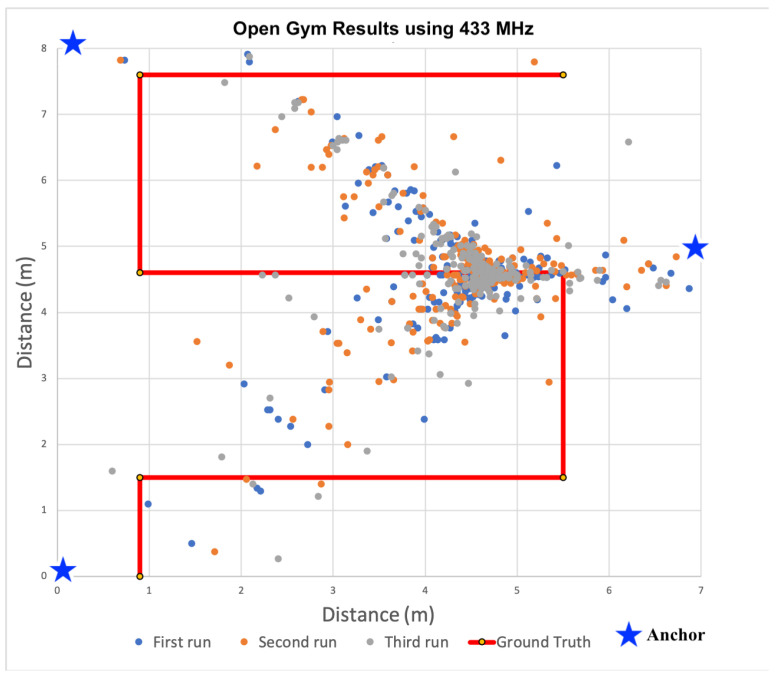
Position estimates obtained using RSS-based localization with 433 MHz transceivers in the University gymnasium. Reference anchor locations used for localization are shown as stars and the solid red line constitutes the ground truth path. The different colored points show the calculated positions while traversing open gymnasium space during multiple successive experiments.

**Figure 10:**
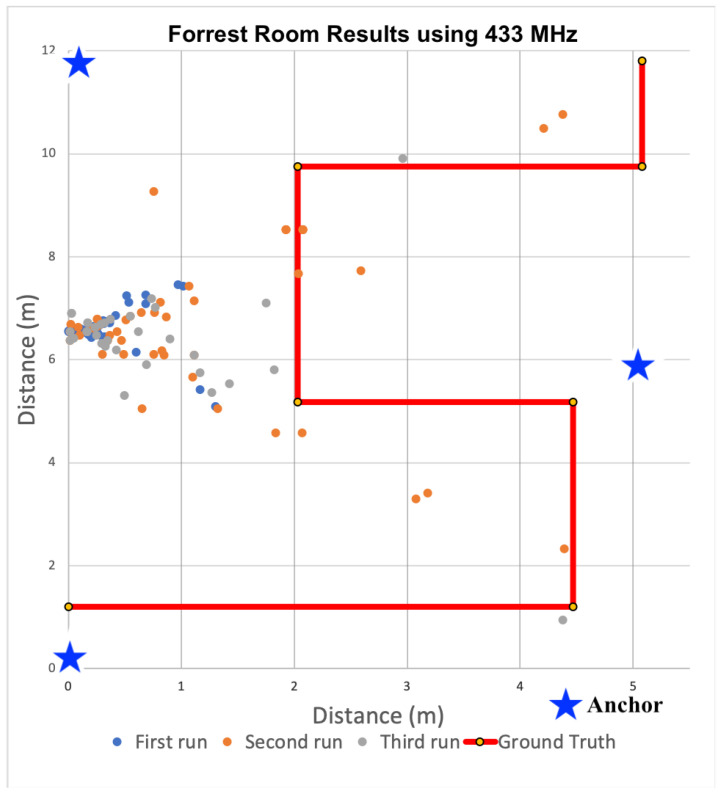
Position estimates obtained using RSS-based localization with 433 MHz transceivers in a mixed use space at the University. Reference anchor locations used for localization are shown as stars and the solid red line constitutes the ground truth path. The different colored points show the calculated positions while traversing the mixed use space during multiple successive experiments.

**Figure 11:**
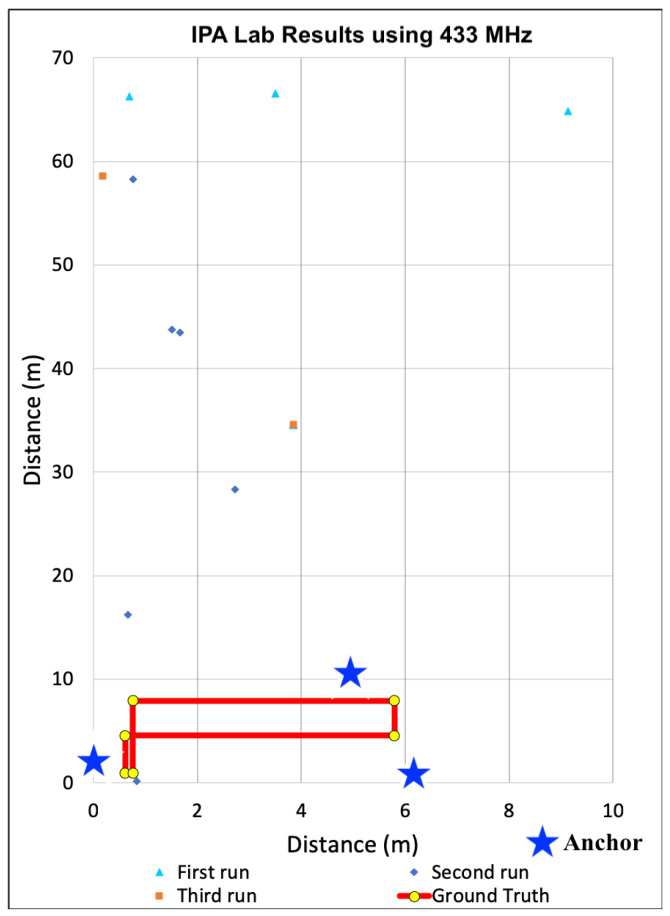
Position estimates obtained using RSS-based localization with 433 MHz transceivers in an engineering lab at the University. Reference anchor locations used for localization are shown as stars and the solid red line constitutes the ground truth path. The different colored points show the calculated positions while traversing the lab during multiple successive experiments.

After analyzing the signal propagation characteristics using 433 MHz transceivers, we built additional anchors devices used the 433 MHz devices to localize within the same three distinct indoor spaces. During all localization experiments, three anchors and a single tag were used to calculate 2D positions. The locations of all anchors were precisely measured. These measurements were configured within the tag firmware and used by each tag for localization calculations. As the tag traversed each space, estimates of received power from transmissions were converted to distances via path loss model and then used to recalculate the tag’s location within the Cartesian plane. To minimize the computational load on each tag, we did not filter the receive power estimates, nor the positions. Only raw, unfiltered values were used to determine positions. These positions are plotted for each indoor space in Figure 9, Figure 10 and Figure 11.

For the results shown in Figure 9, Figure 10 and Figure 11, the solid red line denotes the ground truth path we followed. For each experiment, we placed tape on the ground to ensure we followed the same path consistently for all experiments. We then mounted the tag on a cart to minimize variance in the z-dimension. We also timed the traversal of each leg to ensure each experimental run was completed at a consistent speed. We collected data for at least 10 runs and observed consistent results. Figure 9, Figure 10 and Figure 11 show the calculated positions as a scatter plot as the cart traversed the spaced from three selected runs out of the ten; The different colors depict the positions for the three different traversals. To calculate accuracy and precision, we analyzed the estimated positions for each leg of the path separately relative to the ground truth. We then used the length of each leg to compute the weighted average accuracy, which we then aggregated for the entire path. To characterize position errors, we calculated the euclidean distance of each estimated position relative to ground truth. We then characterized overall precision with respect to the position errors across the entire path.

#### 6.3.1. Localization in Open Gym

The gym was the largest, open space used in our evaluation. Figure 9 shows the estimated positions as a scatter plot for three experimental runs as well as the ground truth path as the solid red line. For this path there were a total of six legs. We followed a serpentine pattern, where each leg was a straight line followed by a 90 degree turn and another traversal in a straight line. This was intended to mimic a search grid pattern an emergency responder might follow to find a victim in zero visibility conditions.

When we analyzed the estimated position data, we found that the weighted average position error was 2.54 m. Looking closer, we found that this varied significantly by leg. For example, in the fourth leg (middle horizontal leg), the average error was only 0.42 m, with a standard deviation of 0.72 m. This is in sharp contrast to the first leg, during which we recorded an average error of 4.62 m with standard deviation of 4.17. We noted that most of the positions appear be clustered around the Cartesian location of (4.5, 4.5) in meters. When the tag is near this location, the accuracy is reasonable and precision is 71.05% at 0.5 m. These results are consistent with our earlier linear ranging results, during which we did not observe proportional changes in estimated distances as the tag was moved away from the anchor. In fact, over a distance of up to 5 m, the estimated distance only changed by 0.4 m during linear ranging. For localization, these low-precision ranges are amplified when used to calculate a position via trilateration. To enable visibility into firefighter locations on the fire ground, this means we would require a significant number of deployed anchors to expect reasonable position estimates even under ideal conditions using 433 MHz transceivers. Moreover, we observed precision of 71.05% for the middle horizontal leg, localization precision of both 0.5 m and one meter for the other legs was either zero or near-zero.

#### 6.3.2. Localization in Mixed Use Space

We next evaluated our localization system within the Forrest Room, an open meeting space with tables and several conference rooms. We chose this space because of it’s similarity with large office buildings. It is notably smaller than the gym and hence more susceptible to multipath effects, which we suspected would negatively impact localization accuracy.

Figure 10 shows the estimated position results during three runs as a scatter plot relative to the ground truth path that followed a similar serpentine path. The results are similar to those from the gym, but with higher estimated position error. Over the entire path, the average weighted error was 3.73 m. Again the paths with the highest average errors were recorded for those located the farthest from the initial calculated positions. These imprecise positions centered around (0, 4.8) meters. Again, these were consistent with our observations during linear ranging as the error increased significantly at higher distances between anchors and tags. The combined effect of using three imprecise ranges further limited positional accuracy. Unlike the gymnasium, the precision of all legs at 0.5 m in this space was near zero. There were a few points that were within the 0.5 or 1 m desired precision targets, but looking more closely at the sequence of positions, these points constituted outliers.

#### 6.3.3. Localization in Engineering Lab

Our final evaluation using 433 MHz was in the IPA research lab. Due to the limited size of the lab, we taped out a circular path that overlapped at the beginning and end. We selected this space due to the potential for high electromagnetic interference due to the electronic equipment and exposed cabling mounted within the confined area. Figure 11 shows the estimated positions as a scatter plot relative to the ground truth. Unlike the results from the other spaces, most of the estimated positions included significant error. This is reflected in our precision estimates as zero position estimates were within either our one meter minimum goal and most of calculated positions were calculated hundreds of meters away. This was due to range estimation inaccuracy resultant to using received power to estimate distance. Over the entire path, the average position error was 307.96 m, which was not possible within the space, rather a direct result of significant range estimation error. In fact, the lowest observed average error was on the top-most horizontal leg at 166.69 m with standard deviation of 84.74. In the worst case, which is shown in the first vertical leg on the left of the graph, the average error was 448.64 m at a standard deviation of 108.96. What this means for firefighters and the incident commanders observing them from outside the building, the estimates would not make it clear that fire teams were within the same block, let alone the same building.

#### 6.3.4. Discussion

Based on the position accuracy results from the experiments in the gym, localization using 433 MHz transceivers seemed feasible for locating firefighters, despite the precision limitations. However, as the scatter plots in Figure 9, Figure 10 and Figure 11 show, the paths taken while traversing the spaces are not discernible in the different environments. Even though the average accuracy error for a path is within 2.54 m in the open-air gym, most of the time the position error is significantly greater. As such, we observe that average accuracy may not be a sufficient measure to enable the level of visibility necessary to track responders. Furthermore, consistency in positions, as captured by precision, is critical.

Based our signal propagation experiments and analysis, we also observed that the type of indoor environment had a significant impact on localization accuracy and precision. Even though this has been shown in previous work, this confirmed the need for techniques that do not require extensive path loss model tuning using a priori knowledge of the space. After collecting and analyzing these results, we considered alternative techniques that did not rely on using received power estimates combined with apriori knowledge embedded within path loss models to estimate distance; we needed an approach that could be more resilient to multipath effects in unknown indoor environments.

### 6.4. Linear Ranging Revisited

We repeated the linear ranging experiments using a symmetric two-way ranging protocol with Ultra Wide Band (UWB) transceivers. Instead of relying on received power estimates, we configured anchor and tags to exchange a series of messages over UWB channels. Time stamps were recorded when messages were sent and upon receipt on both anchors and tags. Distances between devices were then calculated based on the recorded propagation time, also known as time of flight (ToF). We proceeded to repeat the simple linear ranging experiments we did earlier with other transceivers by placing an anchor in a fixed location within an open hallway with no significant obstructions. We then used a device configured as a tag to record multiple estimated distances at incrementally increasing distances from the anchor ranging from one to twelve meters. Although we later did further experiments at longer distances, our goal at this point was to assess distance estimation accuracy using ToF over UWB rather than relying on path loss models with previously tested transceivers.

**Figure 12:**
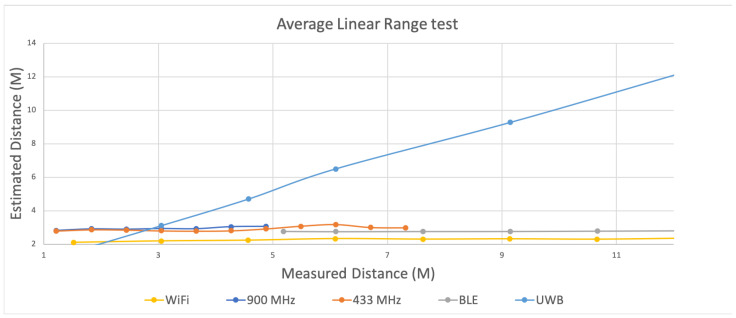
Linear distance estimates relative to measured distance between two devices comparing, BLE, 433 MHz, 915 MHz, Wi-Fi, and Ultra-Wide Band.

Figure 12 compares the earlier linear ranging results using Wi-Fi, BLE, 433 MHz, and 915 MHz with UWB. In an ideal ranging system, estimated distances correlate with actual distances, which is what we observe using UWB. At a close range of 1.52 m, the average estimated distance was 1.56 m with a standard deviation of 0.05 m. When we moved to 12.19 m away from the anchor, the average estimated distance was 12.27 m with a standard deviation of 0.02 m. The largest difference between estimated and measured distance was 0.40 m at 6.09 m. In terms of precision, our analysis showed that 99.81% of the estimated distances were within the required 0.5 m for the firefighting use case. At 6.09 m we did observe a lower precision of 14.2%. We repeated the ranging experiments several additional times at this distance, but the results were consistent. Upon closer inspection we realized that the location was near a steel reinforced, retrofitted wall. When we moved to a different open space, the precision was consistently above 99% out to 12 m. Still, this anomaly did reveal that while UWB is more resilient to multipath effects, these issues still occur. Consequently, this reaffirmed our hypothesis that any indoor localization system evaluation must incorporate a variety of hostile spaces to be considered for use by emergency responders.

### 6.5. Signal Propagation Analysis within Alternative Spaces

After completing linear ranging experiments, we also conducted additional experiments to better understand signal fading within these spaces using ultra-wide band transceivers. We returned to the same three indoor spaces used during earlier localization experiments including the YMCA gymnasium, common study area with attached conference rooms called the Forrest Room, and the IPA research lab, populated with electronic equipment. Unlike the characterization with the 433 MHz transceivers, for these experiments we captured Channel Impulse Response (CIR) accumulator window data during ranging experiments. Within the Decawave UWB transceivers this accumulator window is used to capture the magnitude and time stamp when signals are received. High magnitude peaks are recorded as candidate message arrival times and include those received directly as well as from reflections. Internally, the transceiver selects high magnitude impulses that are most likely to be from a direct path and avoids using those from alternative paths to ensure high accuracy. For line-of-sight cases, which is the case in all of our current localization experiments, peaks with the highest amplitude are typically associated with the direct path, received message time stamp and secondary or tertiary high amplitude peaks constitute reflections.

**Figure 13:**
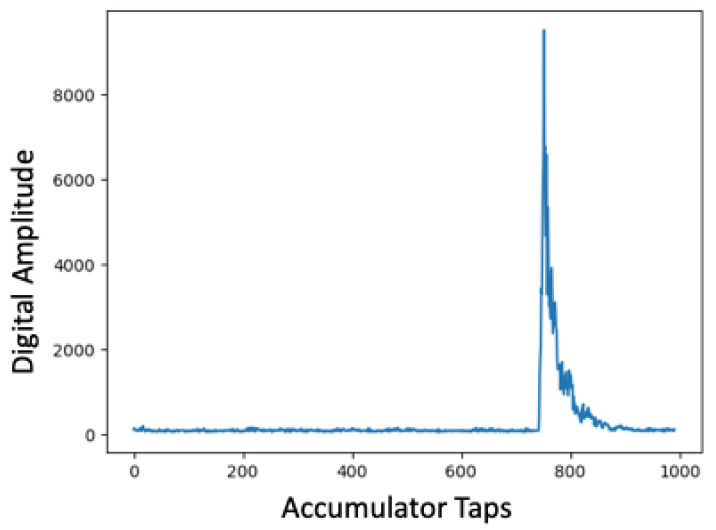
UWB Channel Impulse Response Window captured within the open Gym space. The tail after the high amplitude pulse reveals some reflections within the space, but are limited.

**Figure 14:**
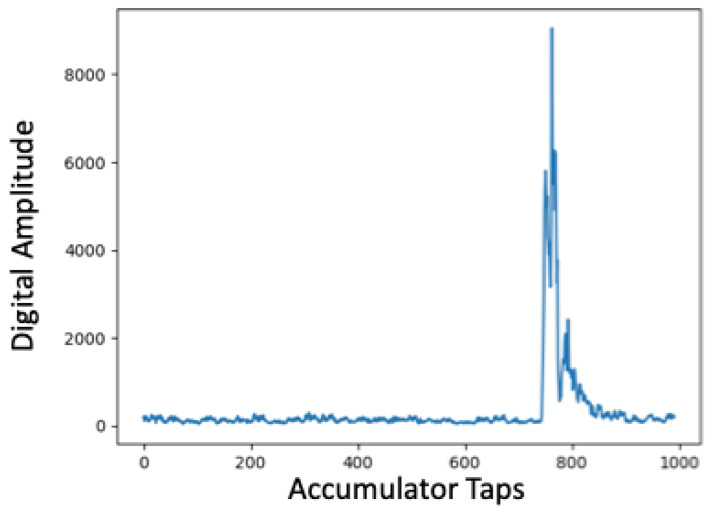
UWB Channel Impulse Response Window captured within the Forrest Room. The accumulator window captured several high magnitude peaks, including one just before the highest peak. These results indicate additional reflections within the space compared to the gym translating into ranging errors and reduced localization accuracy.

**Figure 15:**
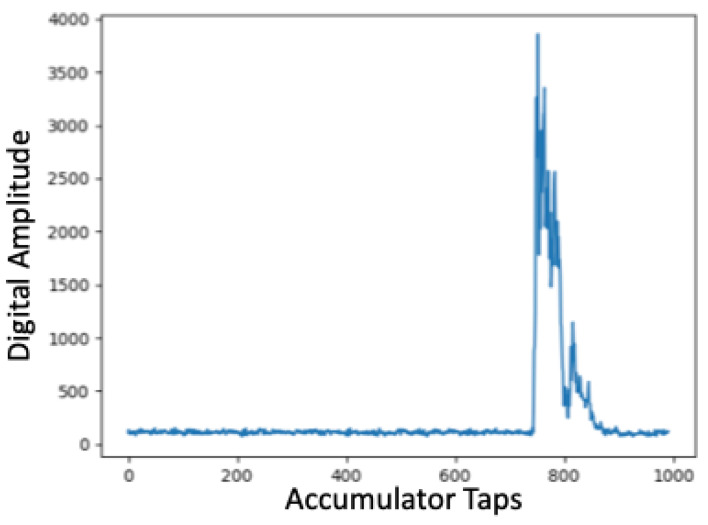
UWB Channel Impulse Response Window captured within the IPA Lab. The accumulator window captured multiple high magnitude peaks. These results indicate significant reflections within the space compared to the gym and Forrest Room translating into a high likelihood of reduced ranging and localization accuracy.

For spaces composed of building materials, obstructions, or other factors that impact wireless signal propagation in the UWB spectrum, CIR window analysis can be used to identify areas within which ranging may be impacted. Figure 13, Figure 14 and Figure 15 show Channel Impulse Response windows captured within the gym, Forrest Room, and IPA lab using UWB at a center frequency of 6.5 GHz. Figure 13 is typical of line-of-sight communication over UWB with a defined, high magnitude pulse. Given the lack of other significant peaks within the accumulator reflects the limited susceptibility of multi-path propagation. The accumulator window in Figure 14 reveals several high magnitude peaks at different accumulator tap points. With a sufficiently high threshold the lower magnitude peaks could be filtered given this was a line-of-sight communication, but in non-line-of-sight conditions in a different space, the difference between the first, lower peak and the higher second peak could amount to ranging error. The fact that there are three distinct peaks in this trace indicates there are additional paths transmitted signals are taking to reach the receiver compared to the gym. Finally, the CIR window from the IPA lab, shown in Figure 15, reveals there are numerous additional paths that transmitted signals are being propagated to the receiver. Similar to our earlier results with 433 MHz transceivers, these UWB results reveal that the material composition and the contents of spaces can have a deleterious impact on signal propagation, impacting ranging and hence localization accuracy.

### 6.6. Localization Using Ultra-Wide Band

After analyzing the impact of the spaces on signal propagation, we then built built several additional anchors using UWB transceivers to analyze the use of UWB for localization within these spaces. Again, three anchors and a single tag were used for all experiments. Similar to the earlier experiments, the locations of all anchors were measured and the firmware for each device was updated with these positions to use within localization calculations. As the tag traversed each space, it was configured to repeatedly exchange ranging messages with each anchor to measure distances from each. These distances were then used to continually recalculate the tag’s location within the Cartesian plane. Even though previous work has leveraged filtering to minimize noise and outliers, in this study, only raw distances were used to determine positions. Moreover, calculated positions were not filtered. To enable further comparison of the impact of accuracy within these spaces, in addition to average error, we also calculated the third quartile of error for each of the traversed paths in each space [62].

**Figure 16:**
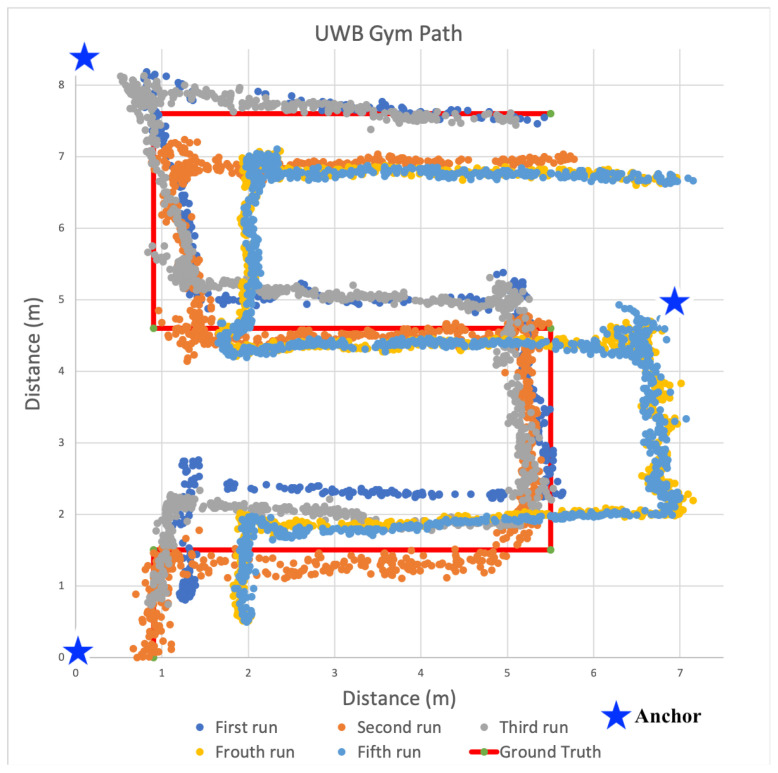
Position estimates obtained using two-way ranging localization with Ultra Wide Band in the open gymnasium space. Reference anchor locations used for localization are shown as stars and the solid red line constitutes the ground truth path. The different colored points show the calculated positions while traversing open gymnasium space during multiple successive experiments.

**Figure 17:**
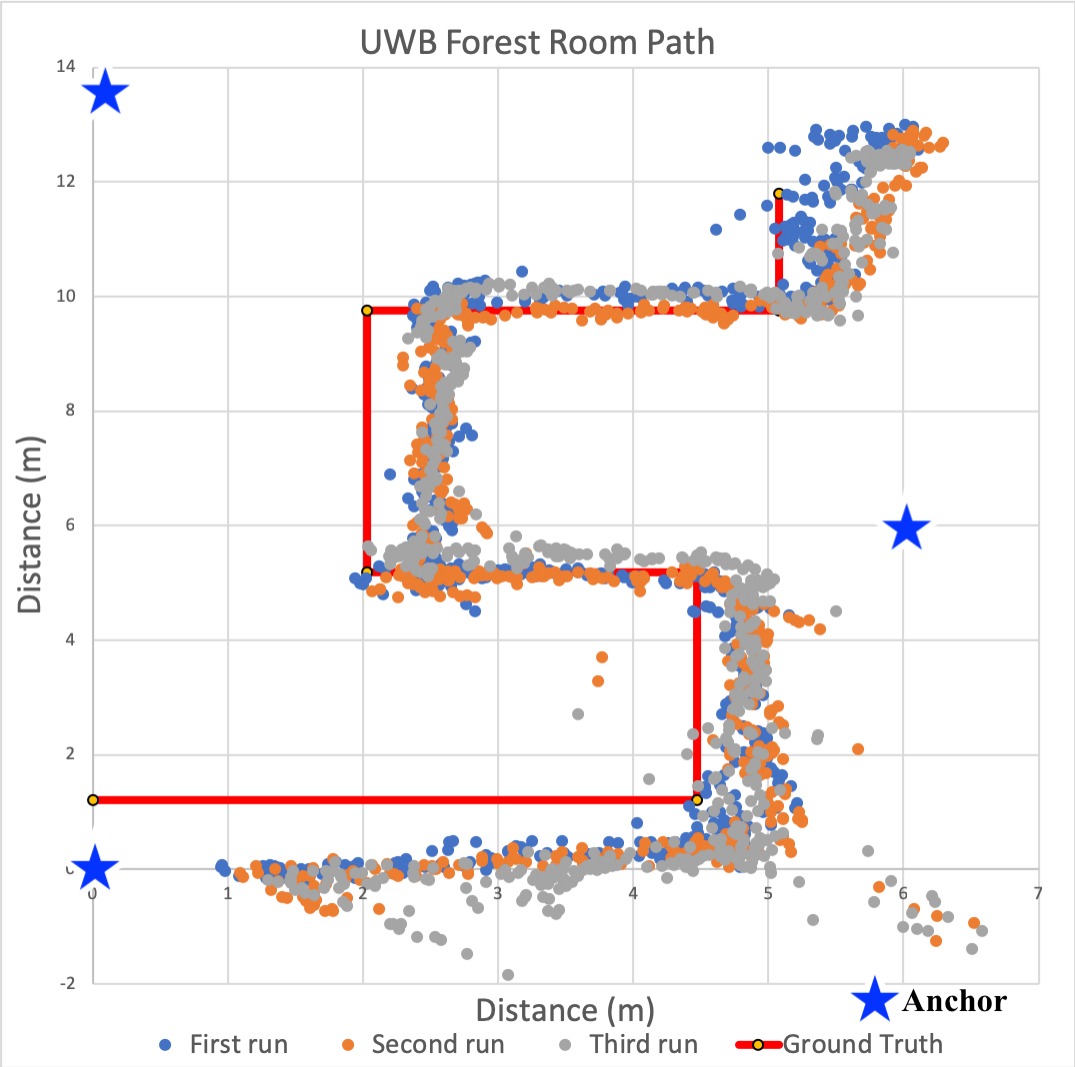
Position estimates obtained using two-way ranging localization with Ultra Wide Band in the mixed use space called the Forrest Room. Reference anchor locations used for localization are shown as stars and the solid red line constitutes the ground truth path. The different colored points show the calculated positions while traversing the Forrest Room space during multiple successive experiments.

**Figure 18:**
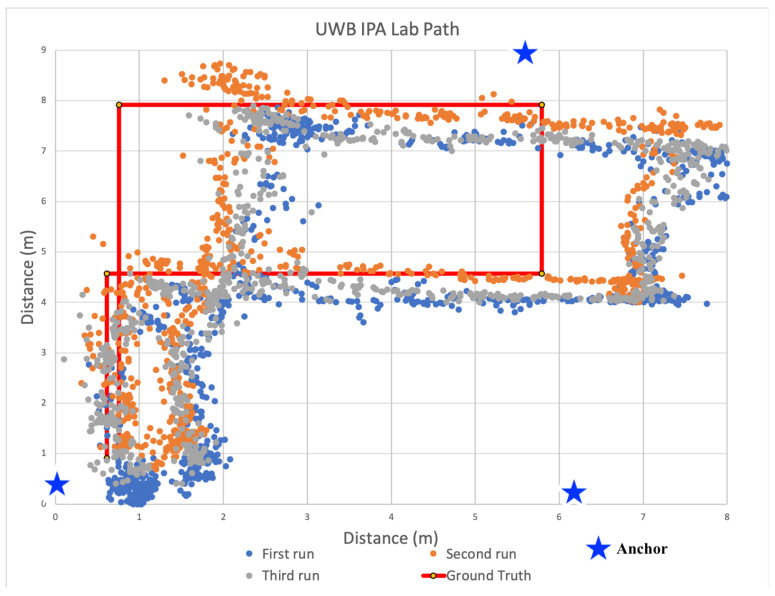
Position estimates obtained using two-way ranging localization with Ultra Wide Band in the IPA research lab space. Reference anchor locations used for localization are shown as stars and the solid red line constitutes the ground truth path. The different colored points show the calculated positions while traversing the IPA lab space during multiple successive experiments.

Figure 16, Figure 17 and Figure 18 show a scatter plot of the localization paths recorded for multiple runs (three to five) in each of the three spaces using UWB-based anchors and a single tag. The red line depicts the ground truth path that was followed within the space and the stars indicate the locations of the anchors. We followed the same serpentine paths as in previous experiments with other transceivers and ensured the anchors were located in the same positions. The scatter plots within each figure show calculated positions as we traversed the ground truth path.

#### 6.6.1. Localization in Open Gym

The position estimates during experiments in the gym are shown in Figure 16. We observed a weighted average error in estimated position of 0.37 m and third quartile error of 0.51 m. Across the path, the last leg (top most horizontal leg) exhibited the highest average accuracy of 0.12 m with a standard deviation of 0.10 m and a third quartile accuracy of 0.21 m. The second leg (lowest horizontal leg) had the lowest average accuracy of 0.83 m with standard deviation of 0.09 m and third quartile accuracy of 0.97 m. We noted that for several of the legs, the estimated positions were consistently skewed, so we also experimented with adjusting the antenna position of the UWB module during ranging. The antenna position adjustments did seem to reduce to position error for some of the legs, but requires further exploration in future work.

The use of Time-of-Flight distance estimation with UWB transceivers led to significantly higher precision in position estimation, even without any adaptive range or position filtering. Across the entire path, one meter precision was 100%, meaning calculated positions were within a single meter. Half meter precision was at 77.095%. What this means is that in best case, line-of-sight conditions within a space that has minimal environmental interference, highly accurate position estimates can be consistently identified using UWB. Additionally, for first responders, the paths taken during each of the experimental runs, shown as orange, blue, and gray scatter plots are clearly visible. While there is some variance in positions and estimated traversal paths between runs, we note that these position estimates do not leverage any filtering on either the ranges or positions over time. In future work we will investigate the use of these techniques to further improve positional accuracy and precision.

#### 6.6.2. Localization in Mixed Use Space

We next moved to the Forrest Room space to evaluate the use of UWB for localization. Three runs of estimated positions calculated while traversing this space are shown in Figure 17. Based on our earlier experiments with 433 MHz devices, we expected a reduction in estimated position accuracy across the path. However, when using UWB-outfitted devices the weighted average error (weighted by leg distance) in estimated position was lower at 0.31 m and 0.47 m in terms of third quartile error. In fact, the average error was only 0.06 m for the fourth leg with a standard deviation of 0.05 m and third quartile error of 0.1 m, while the average error was maximally 0.51 m with a standard deviation of 0.23 m and third quartile error of 0.72 m for the last leg.

In terms of precision, one meter precision was 99.86% and half meter precision was 73.93% across the entire path. Similar to the results from the gym, the path taken is fairly clear and accurate with lower precision within some legs such as the leg near the bottom of the graph. The highest precision are visible with the two horizontal legs near the middle of Figure 17, which had precision rates of 100% at one meter and between 98.27% and 100% at the half meter level. The lowest precision was observed during the lower horizontal leg, where one meter precision was 100%, but half meter precision was 49.35%. This level of accuracy and precision in this space is notable given the building materials and layout are common amongst most commercial office spaces, which are often problematically traversed by firefighters during fire suppression operations. As shown in Figure 17, the paths taken by firefighters in this environment were clearly visible.

#### 6.6.3. Localization in IPA Research Lab

Figure 18 shows a scatter plot of estimated positions recorded while traversing a defined ground truth path within the IPA research lab. Recall that in our previous experiments using received power with 433 MHz transceivers, interference and multipath effects within this space led to localization errors of up to 448.64 m. Using two-way ranging on UWB transceivers, we observed a weighted average position error of 0.56 m and third quartile error of 0.69 m over the entire path. The highest accuracy was observed during traversal of the first leg of the path (bottom left vertical path), with an average position error of 0.19 m, standard deviation of 0.9 m, and third quartile error of 0.25 m. The highest average error occurred during traversal of the third leg (right-most vertical leg), which was 1.25 m, standard deviation of 0.22 m, and third quartile error of 1.38 m, while the estimated positions calculated within this space did exhibit increased error relative to the gym and Forrest Room, the path taken remains discernible when plotted in Figure 18.

The impact of the environment does impact the precision of position estimates as is also visible in Figure 18. Note that calculated positions are noticeably shifted to the right relative to the ground truth path, particularly on the two upper vertical legs. This clearly impacted precision as one meter precision was at 81.87% and half meter precision was at 57.23% overall. In the best cases, which are the horizontal legs, in the upper and lower parts of the path loop, the precision of position estimates was 100% at one meter, but between 85.78% and 94.03% at half meter. In the worst cases, which are shown as the vertical legs in the loop, precision was significantly lower at 6.5% and 68.25% for both one meter and half meter. Still, similar to the other two spaces, the paths taken, while shifted are still clearly visible compared to earlier results using 433 MHz transceivers. For first responders, this still constitutes actionable information during a firefighter rescue scenario, Moreover; these results demonstrate the need to evaluate any indoor positioning system in multiple environments.

#### 6.6.4. Summary and Discussion

Using two-way ranging over UWB improves localization accuracy to within centimeters in most cases. We did observe that the type of space did impact both accuracy and precision as reflected in our signal propagation experiments and analysis. For example, in the Forrest room, the weighted average position error was 0.31 m compared to 0.56 metes within the smaller research lab populated EMI-generating equipment.

One notable difference between ranging with 433 MHz and UWB was the visibility into the paths taken. Unlike the earlier localization results, when we used UWB for ranging the path taken is discernible. For firefighters within a burning structure, this level of visibility is critical. Still, while the engineering research lab did impact the accuracy and precision of position estimates, this evaluation is still limited. For example, the indoor spaces we tested within were dry and composed of typical building materials. To be useful for firefighters, any indoor positioning system would have to operate in more hostile, wet conditions.

### 6.7. Localization at the Fire Training Ground

To evaluate the UWB-based devices in the hostile conditions faced by firefighters, we collaborated with the local municipal fire department to test our simple positioning system in more realistic environments as previously described. Upon arrival to the training facility, we measured each of the spaces and placed anchor devices within resealable bags around the spaces at designated locations. We then conferred with the team on attack tactics to learn the path they would take to traverse the space with a charged fire hose. Based on their description, we marked, measured, and recorded this path to serve as ground truth for later analysis. Before each run we outfitted the fire team with a single tag in a resealable container that they carried through the duration. During each experimental run, the fire team attacked the room with a fire hose delivering water first up the stairs, to the ceiling, then to all walls and floor though a 2″ high pressure fire hose. All devices were configured to send regular heartbeat messages to a server we set up on-scene to ensure functionality through the experiments. This proved critical given several of the devices were blown out of position, or worse out the window, by water from the fire hose.

After the anchors were deployed and configured, we asked the fire team to traverse the spaces several times without the fire hose. Our goal here was to collect baseline localization data in dry conditions, similar to our earlier experiments. We then asked the fire team traverse the space while delivering water to the spaces as they would while battling a fire, while they traversed the space, we collected additional position estimates.

#### 6.7.1. Concrete Tower

In the concrete tower, the rooms were approximately six meters by six meters, and 2.5 m in height, with a staircase on one side that led to the second floor. Within the tower, we used the second floor in our evaluation. This meant that the fire team first directed water up the stairs, entered the room with the devices, and then attacked the room with water from the hose before heading up the stairs to the next floor. Because this evaluation was limited to two dimensions, we did not attempt to localize their movement vertically in the z-dimension.

**Figure 19:**
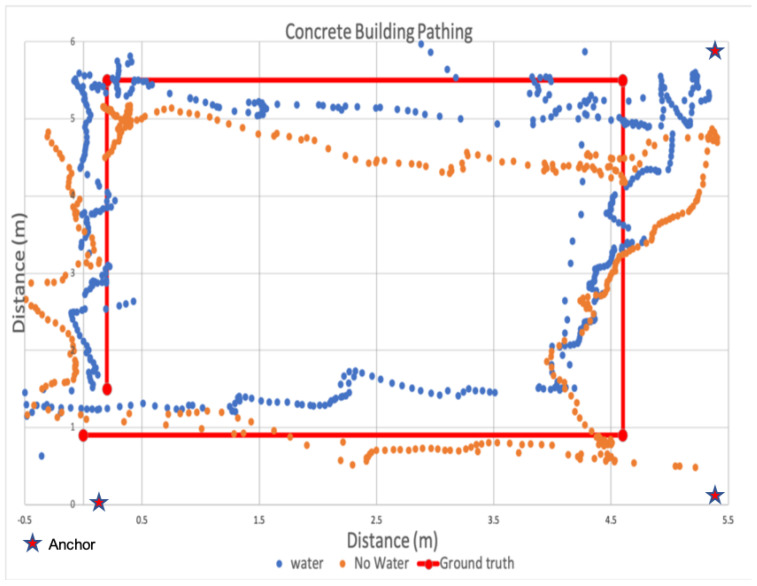
UWB Path testing at Fire Training Facility.

Figure 19 shows a scatter plot of the estimated positions recorded during two runs, one with water and a second without water. The solid red line depicts the ground truth path followed by the fire team. The stars indicate the positions of the anchors, and the two sets of orange and blue points show the estimated positions during wet and dry path traversals. A key goal in this evaluation was to characterize the impact of the extremely wet environment of an active fireground on our localization system, which to the best of our knowledge, has not been previously evaluated. In analyzing this data, we observed there was minimal positional variance between wet and dry environments. In fact, the average estimated position error was actually lower, at 0.38 m with third quartile error of 0.53 m during the traversal when water was being deployed in the space, than our earlier runs, an average error of 1.76 m (with third quartile error of 2.17 m) when the tower was dry. Unfortunately, we could not repeat the dry experiments again that day. However, based on earlier runs, we observed these error differences were within the run to run measurement variance. In other words, the deployment of water did not seem to adversely impact localization accuracy.

We also considered precision for both the dry case as well as the wet case. At the half meter precision level, the overall precision across the path when the tower was dry was 80.82% at one meter and 51.25% at 0.5 m. When water was actively deployed by the hose team, precision changed. Under wet conditions, overall one meter precision was 96.47% and 74.12% at half a meter. This is counter-intuitive, as it implies that precision increases under wet conditions using UWB. However, during later analysis, we found the precision variance between wet and dry runs was within the run to run variance. Consequently, wet conditions did not seem to significantly impact UWB precision. We plan to collect additional data on both accuracy and precision in future visits to the Fire Training Facility. However, these initial results indicate that using UWB within an indoor positioning system designed for firefighters may be practical.

#### 6.7.2. Corrugated Metal Building

**Figure 20:**
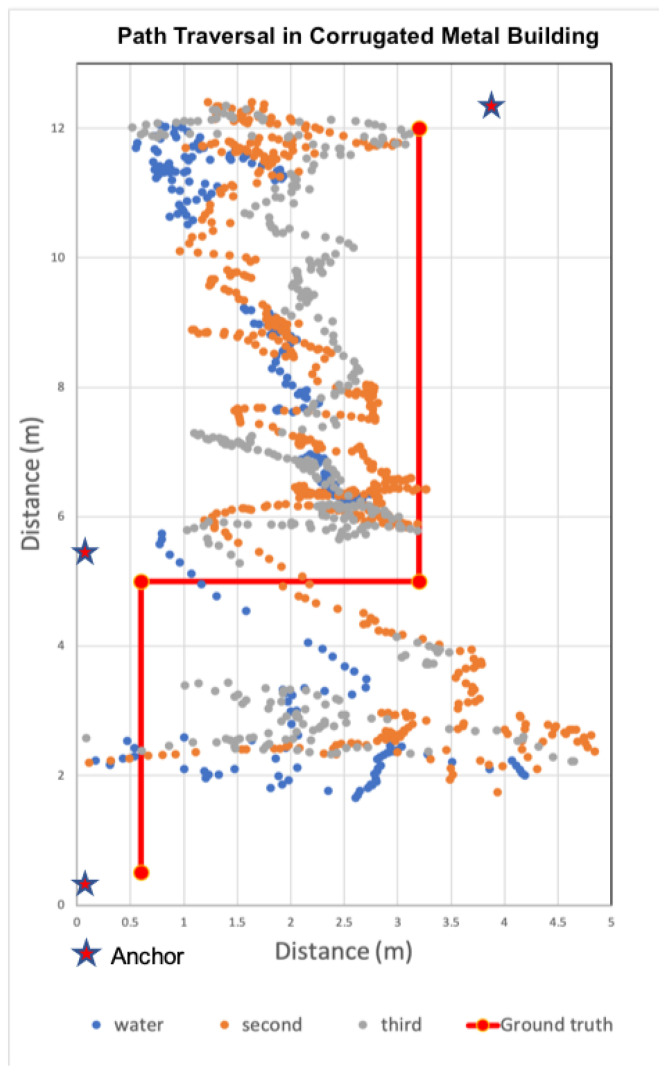
UWB Path testing at Fire Training Facility.

Our final evaluation was within a corrugated metal building constructed of multiple shipping containers that were grouped together with doors in between. As such, the spaces were narrow, with metal stairs located along the sides. We limited our evaluation to the first floor. The composition of this building constitutes a worst case scenario for RF-based localization systems due to the potential for multipath issues [46]. Figure 20 plots the estimated positions recorded during three runs–a wet run and two dry runs. Similar to previous figures, the stars indicate the positions of the three anchors.

The observed accuracy results were unexpected for this indoor space as we hypothesized our UWB-based localization system may not work at all in this difficult environment. The weighted average error over the entire path was 2.83 m and the third quartile error was 3.28 m. The first leg (lower vertical leg) exhibited the lowest average error of 4.33 m, standard deviation of 2.07 m, and third quartile error of 5.6 m. The highest accuracy was observed during traversal of the horizontal leg in the middle. For this leg, the average accuracy was 1.09 m with a standard deviation of 0.92 m and third quartile error of 2.47 m. The impact of water had minimal effect on accuracy.

For all runs with and without water, the metal building had a significant impact on precision. When we tested under dry conditions, we observed 0.5 m was approximately 25.11% overall, ranging from 0% to 38.46% on a per leg basis. When we relaxed the precision to one meter, overall precision across the run was 50.69% and ranged between 37.03% and 93.54%. During runs when water was actively deployed in the space by the hose team, we observed 0.5 m precision was approximately 5% overall, ranging from 0% to 15% on a per leg basis. When we relaxed the precision to one meter in the wet environment, precision ranged between 11% and 17.5%. Based on these results, hostile environments have a clear impact on the accuracy and precision of indoor localization systems, even when using transceivers and ranging protocols that produce high-accuracy positions in non-hostile environments. It should be noted that these results leveraged raw ranges and simplistic lateration techniques for positions estimation. In future work, we anticipate augmenting our geometric localization pipeline to further improve estimates to enable the use of these systems by first responders.

## 7. Discussion and Conclusions

In this paper, we characterized the feasibility of leveraging several common transceivers and ranging techniques to build an indoor localization system specifically for locating and tracking firefighters within structures during building fires. Based on our linear ranging experiments, we found that even when coupled with tuned path loss models for distance estimation, the most commonly used transceivers today, including Wi-Fi, BLE, 915 MHz, and 433 MHz, do not accurately identify distances without techniques that rely on significant a priori knowledge of the space characteristics. Without accurate range estimates estimating positions using multiple anchors via lateration is error-prone, and only gets worse with within hostile environments faced by firefighters. Our results showed that even with the most promising linear ranging results, as was the case using 433 MHz transceivers, building a localization system with these is impractical for first responders.

Although commonly used transceivers failed to provide sufficient ranges to accurately localize indoors, the use of a symmetric two-way ranging protocol with ultra-wide band transceivers did provide ranges with minimal errors in most environments, resulting in a significant improvement in position estimation. Our results showed that across three different common spaces, including a large, open gymnasium, mixed office space, and an electrical engineering laboratory, we were able to consistently localize a single tag using a simple lateration-based system. We quantitatively showed that even without using more advanced localization techniques, our initial system could accurately track public safety personnel in a variety of space types. Our experimental results also showed that the composition of indoor spaces has a direct impact on both accuracy and precision, while this is intuitive, evaluation of localization systems within the hostile environments faced by firefighters has only been minimally explored previously.

Although these results show promise for using RF-based localization systems by first responders, our evaluations with local fire departments show that there are still many opportunities for improvement. For example, in the future we intend to incorporate additional filtering techniques into both the ranging and position estimation parts of our localization pipeline to minimize transient ranging errors. We also plan to incorporate an optimization-based approach to calculate positions with distance estimates from more than three anchors. Additionally, while our results show promise for tracking firefighters during emergency events, our current system limits the number of tags that can be tracked concurrently. We plan to further explore techniques to improve position estimation for dynamic environments as well as scale our system for use by firefighters.

## Data Availability

Not applicable.
